# Virus and Host Factors Affecting the Clinical Outcome of Bluetongue Virus Infection

**DOI:** 10.1128/JVI.01641-14

**Published:** 2014-09

**Authors:** Marco Caporale, Luigina Di Gialleonorado, Anna Janowicz, Gavin Wilkie, Andrew Shaw, Giovanni Savini, Piet A. Van Rijn, Peter Mertens, Mauro Di Ventura, Massimo Palmarini

**Affiliations:** a Istituto Zooprofilattico Sperimentale dell'Abruzzo e Molise G. Caporale, Teramo, Italy; b MRC-University of Glasgow Centre for Virus Research, Glasgow, United Kingdom; c Central Veterinary Institute of Wageningen University, Department of Virology, Wageningen, The Netherlands; d Department of Biochemistry, North-West University, Potchefstroom, Republic of South Africa; e The Pirbright Institute, Pirbright, United Kingdom

## Abstract

Bluetongue is a major infectious disease of ruminants caused by bluetongue virus (BTV), an arbovirus transmitted by Culicoides. Here, we assessed virus and host factors influencing the clinical outcome of BTV infection using a single experimental framework. We investigated how mammalian host species, breed, age, BTV serotypes, and strains within a serotype affect the clinical course of bluetongue. Results obtained indicate that in small ruminants, there is a marked difference in the susceptibility to clinical disease induced by BTV at the host species level but less so at the breed level. No major differences in virulence were found between divergent serotypes (BTV-8 and BTV-2). However, we observed striking differences in virulence between closely related strains of the same serotype collected toward the beginning and the end of the European BTV-8 outbreak. As observed previously, differences in disease severity were also observed when animals were infected with either blood from a BTV-infected animal or from the same virus isolated in cell culture. Interestingly, with the exception of two silent mutations, full viral genome sequencing showed identical consensus sequences of the virus before and after cell culture isolation. However, deep sequencing analysis revealed a marked decrease in the genetic diversity of the viral population after passaging in mammalian cells. In contrast, passaging in Culicoides cells increased the overall number of low-frequency variants compared to virus never passaged in cell culture. Thus, Culicoides might be a source of new viral variants, and viral population diversity can be another factor influencing BTV virulence.

**IMPORTANCE** Bluetongue is one of the major infectious diseases of ruminants. It is caused by an arbovirus known as bluetongue virus (BTV). The clinical outcome of BTV infection is extremely variable. We show that there are clear links between the severity of bluetongue and the mammalian host species infected, while at the breed level differences were less evident. No differences were observed in the virulence of two different BTV serotypes (BTV-8 and BTV-2). In contrast, we show that the European BTV-8 strain isolated at the beginning of the bluetongue outbreak in 2006 was more virulent than a strain isolated toward the end of the outbreak. In addition, we show that there is a link between the variability of the BTV population as a whole and virulence, and our data also suggest that Culicoides cells might function as an “incubator” of viral variants.

## INTRODUCTION

Bluetongue is one of the major infectious diseases of ruminants and is caused by bluetongue virus (BTV), a virus transmitted from infected to uninfected hosts by Culicoides biting midges (1). BTV is the type species of the genus Orbivirus within the virus family Reoviridae and possesses a genome consisting of 10 segments of double-stranded RNA (dsRNA) encoding 7 structural and 4 nonstructural proteins (1–3). The icosahedral particle is organized as a triple layer of capsid shells (4, 5). The outer capsid is formed by VP2 and VP5, while the inner layer is composed of two major proteins, VP3 (subcore) and VP7 (core), encasing the 10 genomic segments of linear dsRNA and three minor enzymatic proteins, VP1 (RNA-dependent RNA polymerase), VP4 (RNA capping enzyme), and VP6 (RNA-dependent ATPase and helicase) (2, 4, 5). In addition, BTV expresses four nonstructural proteins (NS1, NS2, NS3, and NS4) involved in virus replication and morphogenesis and in counteracting the innate immune system of the host (3, 6, 7).

There are at least 26 BTV serotypes (BTV-1 to BTV-26) circulating worldwide. Serotypes are determined primarily by differences in the outer capsid protein VP2, which induces neutralizing antibodies in infected animals (8–13).

Bluetongue is enzootic in areas where the mammalian reservoirs, the virus, and the insect vector have the opportunity to coexist in climatic conditions conducive to BTV replication and transmission. As a result, historically BTV was present exclusively in tropical and subtropical areas of the world, where suitable conditions exist. However, in the last 10 to 20 years, the global distribution of bluetongue, similarly to some of the other vector-borne diseases, has expanded dramatically, potentially due to a variety of factors, including an increased global travel and commerce, deforestation, and climate change (14–17).

An interesting aspect of bluetongue is the extreme variability of the clinical outcome as a result of BTV infection. In many cases, BTV induces only mild or inapparent clinical infections, while in others it can kill the infected host. Symptoms of bluetongue have been attributed mainly to the damage of small blood vessels increasing vascular permeability and resulting in hyperemia, congestion, vascular thrombosis, localized/diffused edema, hemorrhages, and erosion of the mucous membranes. The main clinical signs of affected animals include fever, depression, respiratory distress, and anorexia (18–21).

This variability of clinical outcomes induced by BTV has been attributed to a variety of factors, such as species, breed, age, and the immune status of the mammalian host, as well as the serotype/strain of the virus (21–24). In general, sheep, yak, llamas, and alpacas have been described as the most sensitive species to BTV-induced disease. Cattle and other wild ruminants have a certain degree of resistance to disease, although they are fully susceptible to infection. Cattle show longer periods of viremia and are, therefore, considered reservoirs of infection (21, 25–31). Goats are also susceptible to BTV infection but do not appear to be very susceptible to disease, although contrasting reports appear in the literature, and the heterogeneous experimental conditions used in different studies make it difficult to compare the available data (19, 24, 32–36).

The immunologic status of infected animals understandably has a major influence on the susceptibility to infection and explains why outbreaks of bluetongue typically occur when susceptible animal species are introduced into areas where BTV is endemic or when virulent strains of BTV reach previously unexposed ruminant populations (21). Animals infected with a specific BTV serotype produce long-lasting neutralizing antibodies with limited cross-protection against heterologous serotypes (37). Environmental factors, such as the exposure to solar radiation or high temperatures, can also exacerbate the disease symptoms (38, 39).

While infection of sheep in the tropics and subtropics is common, clinical disease in indigenous breeds is rarely observed. The North European breeds of sheep have been described to be very susceptible to BTV-induced disease as opposed to African or South-East Asian breeds (19, 22, 40–47). Within the same sheep breed, or even within the same flock, there may be considerable differences in the severity of the disease occurrence in individual animals (21, 23).

Serotypes/strains of BTV with different degrees of virulence have been described in the literature. For example, the North European BTV-8 strains that spread since 2006 in Northern Europe is considered highly virulent, as it induced high levels of mortality in naive sheep and in some cases also caused severe clinical disease in cattle (48–51). On the other hand, it is interesting to note that no clinical cases of disease were observed even in sheep when BTV-8 reached Northern Italy and Sardinia a few years later (G. Savini, personal communication). Other serotypes related to vaccine strains (BTV-6, BTV-11, BTV-14) have entered Europe briefly, in general showing very little pathogenicity in the field (52–54).

Bluetongue is experimentally reproducible, and several studies have addressed, directly or indirectly, the variability of the clinical outcome resulting from BTV infection, although at times with contradictory results (55, 56). The heterogeneous experimental conditions used in different studies make it difficult at times to compare the available data. For example, many of the BTV strains used in experimental studies have been passaged more or less extensively in cell culture, and this can potentially lead to attenuation of virulence (57, 58). In addition, some reports in the literature stress that experimental infection using BTV strains isolated in mammalian cell cultures from lethal cases of bluetongue most often results only in the induction of mild clinical signs of the disease (39, 59, 60). Thus, some investigators have used blood from viremic animals as an inoculum, and this appeared to be a very effective way to induce severe clinical signs in the infected animals (20, 61). However, the induction of severe clinical signs of bluetongue have also been reported using BTV passaged in cell culture (62) or virus isolated in embryonated eggs (32, 40).

Here, we used a single experimental framework and standardized conditions in order to systematically assess virus and host factors influencing the clinical outcome of BTV infection. We evaluated differences in susceptibility to BTV-induced disease in goats and sheep of different breeds. In addition, we studied differences in the virulence of two divergent BTV serotypes (BTV-2 and BTV-8), as well as the virulence of different BTV-8 strains isolated at the beginning and end of the North European outbreak of 2006 to 2008. Finally, we evaluated whether genetic bottlenecks (63) exist that can influence BTV adaptation in Culicoides and mammalian cells and also how these influence virulence.

## MATERIALS AND METHODS

### Cells.

Mammalian cells were grown at 37°C in a humidified atmosphere supplemented with 5% CO_2_. BHK-21, BSR (a clone of BHK-21 cells), and African green monkey Vero cells were grown in Dulbecco's modified Eagle's medium (DMEM) supplemented with 10% fetal bovine serum (FBS). CPT-Tert cells are sheep choroid plexus cells immortalized with the simian virus 40 (SV40) T antigen and human telomerase reverse transcriptase (hTERT) and were grown at 37°C in Iscove's modified Dulbecco's medium (IMDM), supplemented with 10% FBS (64). KC cells (65) were derived from Culicoides sonorensis larvae and grown at 28°C in Schneider's insect medium supplemented with 10% FBS.

### Virus strains and titrations.

BTV-8_NET2006_ (Pirbright reference collection number NET2006/04) was originally isolated from a naturally infected sheep during the 2006 outbreak in Northern Europe and has been previously described (3). BTV-8_NET2007(blood)_ was derived from the spleen of a sheep infected with blood derived from a naturally infected cow in the Netherlands during the 2007 BTV-8 outbreak as already described (66). Further viruses were isolated *in vitro* from BTV-8_NET2007(blood)_ after (i) 1 passage in KC cells [BTV-8_NET2007(1KC)_], (ii) 1 passage in KC and 1 passage in BHK_21_ cells [BTV-8_NET2007(1KC-1BHK)_], and (iii) 1 passage in KC and 2 passages in BHK_21_ cells [BTV-8_NET2007(1KC-2BHK)_].

BTV-2_IT2000_ and BTV-8_IT2008_ were derived from naturally occurring outbreaks of bluetongue in sheep in Italy and were isolated in 2000 and 2008, respectively. All viruses used in this study were isolated in KC cells and subsequently passaged twice in BHK-21 cells before use in experimental infections. Virus stocks were prepared by infecting BHK-21 cells at a multiplicity of infection (MOI) of 0.01 and collecting the supernatant when obvious cytopathic effect (CPE) was observed. Supernatants were clarified by centrifugation at 500 × *g* for 5 min, and the resulting virus suspensions were aliquoted and stored at 4°C. Virus titers were determined by standard plaque assays (67). In order to compare the growth of the various BTVs strains used in this study, CPT-Tert cells were infected at an MOI of 0.01, and supernatants were collected at 8, 24, 48, 72, and 96 h postinfection (p.i.). Samples from each time point were subsequently titrated by endpoint dilution analysis in BSR cells, and titers were expressed as 50% tissue culture infective doses (TCID_50_). Each assay was repeated at least twice using two different virus stocks.

### BTV genome sequencing.

The complete genome sequences were derived from the following strains: BTV-8_IT2008_, BTV-8_NET2007(blood)_, BTV-8_NET2007(1KC)_, BTV-8_NET2007(1KC-1BHK)_, and BTV-8_NET2007(1KC-2BHK)_. dsRNA was extracted from the spleen or infected cells as previously described (57). Full-length genome segments were amplified from dsRNA using the SuperScript III One-Step reverse transcription (RT)-PCR system with Platinum *Taq* DNA polymerase (Invitrogen) using primers complementary to the 5′- or 3′-end terminus of the viral genome segments. The genome of BTV-8_IT2008_ was sequenced using the Sanger method. For the other viruses, equimolar, purified PCR products of the 10 genomic segments of each virus were pooled and sheared by focused sonication (Covaris), followed by size selection using Ampure XP magnetic beads. Illumina MiSeq libraries were generated using the KAPA real-time library preparation kit (KAPA), further quantified using quantitative RT-PCR (qRT-PCR; KAPA), and sequenced using an Illumina MiSeq with a 300-cycle cartridge as suggested by the manufacturers. Analysis of genetic diversity was carried out using CLC Genomic Workbench version 6.0.1 (CLC bio). After quality assessment and the removal of sequencing artifacts, reads were mapped using BTV-8_NET2006_ as a reference sequence, and the consensus sequences were extracted. Reads with a similarity fraction below 70% were omitted in the final assembly. Single nucleotide polymorphisms were identified using the quality-based variant detection function within CLC Genomics Workbench version 6.0.1. Total sample reads were mapped to the consensus sequence of each segment, and variants were called using, as parameters, nucleotides with total coverage of over 100 reads and a central quality score of Q20 or higher. Average quality score per nucleotide was above Q35.8 in all samples. The mean depth of coverage per variant in each viral genome was between 8,154 and 12,461. Presence of both forward and reverse reads was required to call a variant, while the frequency threshold was arbitrarily set at 0.1%.

### Experimental infections in mice.

Transgenic mice deficient in type I interferon (IFN) receptor (129sv IFNAR^−/−^; B&K Universal Ltd.) were maintained at biosafety level 3. For each experiment, groups of adult mice matched for sex and age (*n* = 5 per group) were infected intraperitoneally with 300 PFU of virus or mock infected as indicated in Results. Mice were examined for clinical symptoms daily until the experiment was concluded at 14 days postinfection.

### *In vivo* pathogenicity studies.

Animal experiments were carried out at the Istituto Zooprofilattico Sperimentale dell'Abruzzo e Molise “G. Caporale” (Teramo, Italy) in accordance with locally and nationally approved protocols regulating animal experimental use (protocol no. 10933/2011 and 7440/2012). Studies were conducted using a total of 65 sheep and 10 goats held in an insect-proof isolation unit with veterinary care. All animals were confirmed to lack antibodies toward BTV using a BTV blocking enzyme-linked immunosorbent assay (ELISA) as previously described (68). The absence of BTV-specific antibodies was confirmed for each animal using a BTV-specific qRT-PCR in blood samples (see below). For this study, all animals were infected intradermally with a total of 2 × 10^6^ PFU (in 5 ml) of the specific BTV strains indicated below by multiple inoculations in the inner leg and in the prescapular areas. Negative controls were inoculated with 5 ml of mock-infected cell supernatant. Groups (*n* = 5 animals per each group) of domestic goats, 8-month-old Dorset and 2-year-old Sardinian, Dorset, and Italian mixed-breed sheep, were infected with BTV-8_NET2006_. Two additional groups of Sardinian sheep were inoculated with BTV-8_IT2008_ or BTV-2_IT2000_. Two additional groups of Sardinian sheep (*n* = 5 per group) were inoculated with either 5 ml of infected blood [BTV-8_NET2007(blood)_] or with the same virus after passage in KC and BHK_21_ cells [BTV-8_NET2007(1KC-2BHK)_]. All viruses used in this study have the same passage history (1 passage in KC cells and two passages in BHK_21_ cells) unless indicated otherwise. Five goats and 25 sheep (5 adult Dorset, 5 young Dorset, 5 Italian mixed-breed, and 10 Sardinian sheep) were used as negative controls and were inoculated with uninfected cell culture media. Blood samples were collected (with EDTA) from all infected animals daily for 15 days postinfection and thereafter at days 17, 19, 21, and 28 p.i., when the experiment was concluded. The blood samples were analyzed for the presence of viremia by qRT-PCR (see below). Serum samples were collected from each animal on the day of the inoculation (day 0) and then at days 7, 14, 21, and 28 p.i. Sera were tested by virus neutralization assay for the presence of BTV-specific antibodies. Body temperature and clinical signs were recorded daily, beginning a week before inoculation, until day 15 p.i. and subsequently at days 17, 19, 21, and 28 pi. Fever was defined as rectal temperature above 40°C. Clinical signs were scored using a clinical reaction index (CRI) with minor modifications as already described (66) (see Table S1 in the supplemental material).

### Virus neutralization assays.

The presence of neutralizing antibodies in infected sheep and goats, against the BTV strains used, was assessed by neutralization assays testing serial 2-fold dilutions of sera as already described (69). Briefly, serum dilutions (1:10 to 1:1,280) and a fixed amount of virus (100 TCID_50_) were incubated for 1 h at 37°C in 96-well plates, whereupon a 100-μl suspension of Vero cells (3 × 10^5^/ml) was added to each well in minimum essentials medium (MEM). Plates were incubated for 6 to 7 days at 37°C, 5% CO_2_, after which monolayers were then scored for cytopathic effect (CPE). The titer of neutralizing antibodies in each serum sample was determined by endpoint dilution assays (70). Values reported for each sample are the log_10_ of the 50% endpoint (proportionate distance [PD]) from 4 replicates performed using VERO cells.

### qRT-PCR.

Viremia in experimentally infected animals was assessed by qRT-PCR as already described (57, 69). Briefly, blood samples (500 μl) were pretreated with 1 ml cold distilled water on ice for 10 min and then centrifuged at 4°C for 10 min at 13,000 × *g*. Armored RNA (Asuragen, USA) was added to each sample before RNA extraction and used as an internal control to verify RNA extraction efficiency. Total RNA was extracted from the resulting cellular pellet, using the High Pure nucleic acid extraction kit (Roche, Nutley, NJ), in accordance with the manufacturer's instructions. The quality of the samples was further assessed by amplifying the sheep β-actin gene as previously described (71). For each sample, 250 ng of RNA was used in a one-step qRT-PCR employing primers/probes for segment 5 (encoding NS1) of BTV and the armored control RNA. Samples were analyzed using a 7900HT fast real-time PCR system and the sequence detection system software SDS, version 2.3 (Applied Biosystems). BTV genome copy numbers expressed as log_10_/μg of total RNA were derived using a standard curve generated from the amplification of *in vitro* transcribed synthetic BTV segment 5 RNA using the mMESSAGE mMACHINE T7 Ultra kit (Ambion), according to the manufacturer's instructions. Signal levels with threshold cycle (*C_T_*) values of ≥40 were considered negative.

### Statistical analysis.

Statistical analysis was carried out using the software Prism (GraphPad). Significance of differences in body temperature between groups of infected animals was estimated by calculating the total area under the curve (AUC) of body temperatures between days 3 and 11 p.i. for each animal. Significant differences between groups were calculated using an unpaired *t* test or analysis of variance (ANOVA) as appropriate. The AUC relative to the levels of BTV RNA in the blood was calculated for each animal from day 1 p.i. to the end of the experiment, and groups were compared using an unpaired *t* test or ANOVA as appropriate. In addition, significant differences in the peak levels of viremia were also compared using an unpaired *t* test or ANOVA as appropriate.

### Nucleotide sequence accession numbers.

Sequences of BTV-2_IT2000_ and BTV-8_IT2008_ have been deposited in GenBank and were assigned accession numbers KM053268 to KM053277 (BTV-2_IT2000_) and KM053258 to KM053267 (BTV-8_IT2008_). The raw data used for deep-sequencing analyses are available upon request.

## RESULTS

### Replication kinetics *in vitro* and virulence in mice of BTV-2_IT2000_, BTV-8_NET2006_, and BTV-8_IT2008_.

In order to investigate virus and host factors affecting the clinical outcome of BTV infection, we initially focused on three different strains of bluetongue: a BTV-2 strain isolated from Italy in 2000 (BTV-2_IT2000_), a BTV-8 strain isolated from the Netherlands in 2006 (BTV-8_NET2006_), and a BTV-8 strain isolated in Italy in 2008 (BTV-8_IT2008_).

**FIG 1:**
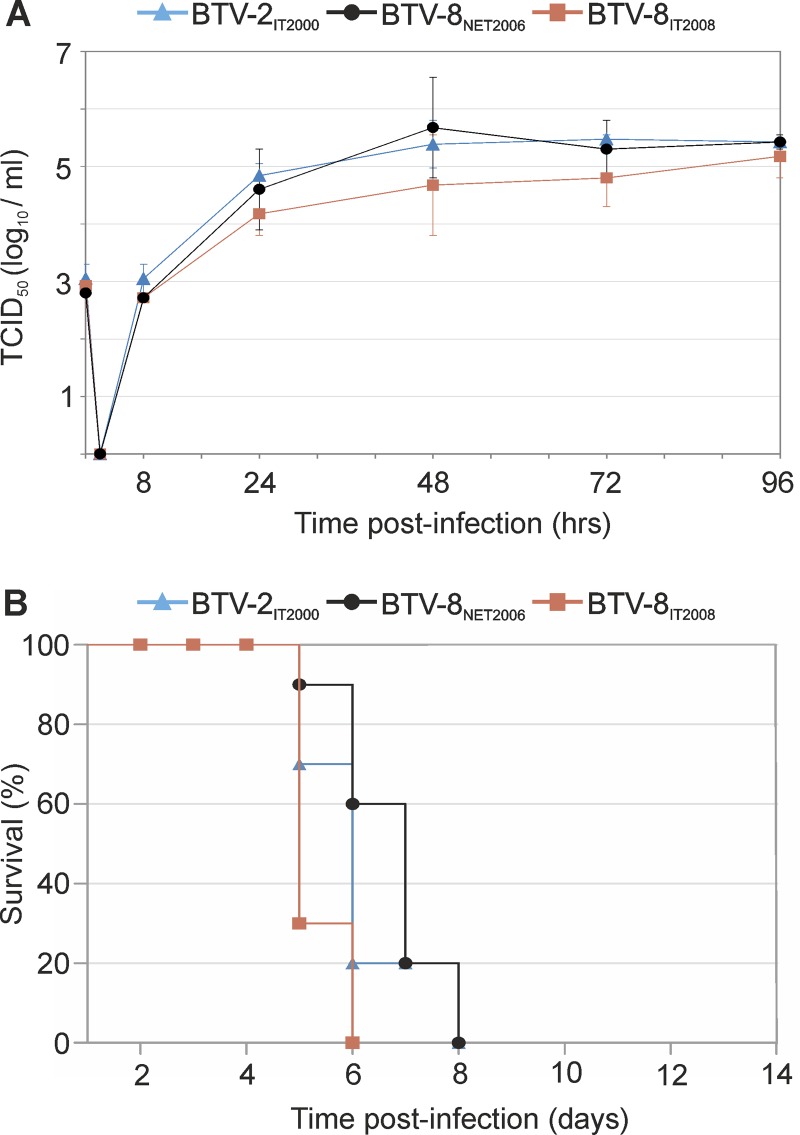
*In vitro* replication kinetics and pathogenicity in mice of the BTV strains used in this study. (A) Replication kinetics of BTV-2_IT2000_, BTV-8_NET2006_, and BTV-8_IT2008_ in sheep CPT-Tert cells. Cells were infected at a multiplicity of infection (MOI) of 0.05, and supernatants were collected 8, 24, 48, 72, and 96 h postinfection. Supernatants were then titrated in BSR cells by limiting dilution assays. Experiments were repeated independently three times, and data are represented as averages from the experiments. Error bars indicate standard errors. (B) Survival plots of 129sv IFNAR^−/−^ mice inoculated intraperitoneally with 300 PFU of BTV-2_IT2000_, BTV-8_NET2006_, and BTV-8_IT2008_. Mice were observed for 2 weeks postinoculation for the presence of clinical signs of systemic disease. All the viruses used in this study killed all the infected mice between days 6 and 8 postinoculation. None of the five mock-infected mice showed any clinical symptoms (not shown in the figure) and survived throughout the observation period.

First, we assessed the ability of all viruses to replicate in sheep CPT-Tert cells. No major differences were observed in the replication kinetics of the viruses regardless of the serotype and strain used in the assay (Fig. 1A). We next assessed the virulence of each strain in IFNAR^−/−^ mice, as these mice succumb to wild-type BTV infection (57, 72). Mice were inoculated intraperitoneally with 300 PFU of the BTV strains described above. All of the mice inoculated with the various BTV strains showed clinical signs around 3 days p.i., characterized by ocular discharge, apathy, and lethargy. All BTV-infected mice died between 6 and 8 days postinfection, while no signs of disease were observed in the control mock-infected mice (Fig. 1B).

### Influence of species, breed, and age of the mammalian host on the clinical outcome of BTV infection.

Several studies investigating the factors that affect the clinical outcome to BTV infection have already been published (1, 20, 21, 73). Here, we aimed to assess the variables affecting the pathogenesis of bluetongue in a single experimental framework. First, we assessed the outcome to BTV infection in 2-year-old goats and sheep of three different breeds (the Northern European Dorset poll, the Italian Sardinian sheep, and a mixed breed from Central Italy). An additional group of Dorset poll sheep, 8 months old in age, were also used in the study. We deliberately used viruses isolated in KC cells and subsequently passaged twice in BHK-21 for all the experimental infections carried out in this study. This strategy allowed us to use viruses minimally passaged *in vitro* and with the same history in cell culture.

**FIG 2:**
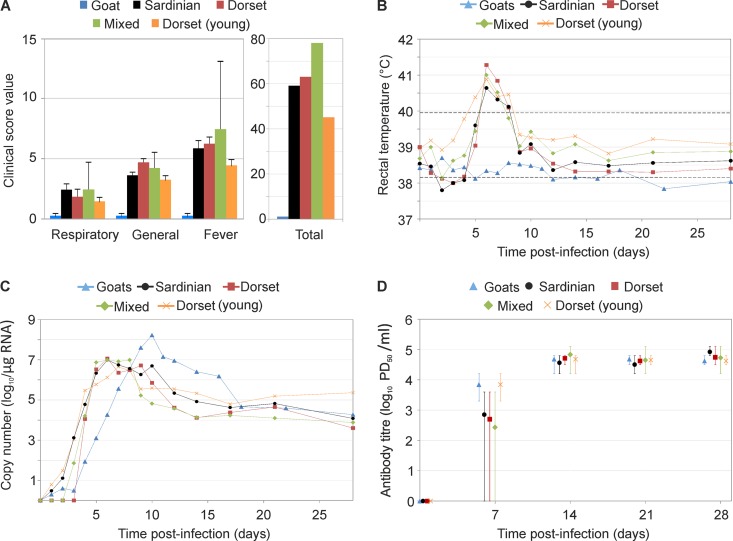
Experimental infection of goats and different sheep breeds with BTV-8_NET2006_. (A) Graphs showing clinical signs recorded in BTV-infected goats and various sheep breeds, including Sardinian, mixed breed, and Dorset poll (*n* = 5 per each group). Animal were all approximately 2 years of age with the exception of an additional group of 8-month-old Dorset poll sheep that are indicated as “Dorset (young).” Animals were scored daily after infection using a clinical index score (shown in Table S1 in the supplemental material), taking into account general symptoms, respiratory signs, fever, need for veterinary intervention, or death. General symptoms included are depression, anorexia, and facial and feet lesions. Each group of 5 animals was infected with the same dose of BTV-8_NET2006_ intradermally. Scores shown for respiratory symptoms, general symptoms, and fever represent the average values collected for each group (±standard error) during the duration of the entire experiment (28 days). Total scores are instead the cumulative values for each symptom within a group collected throughout the observation period. (B) Body temperature (average per group; values per each individual animal are shown in Fig. S1 in the supplemental material) of animals infected with BTV-8_NET2006_. Physiological temperature in sheep ranges normally between 38.3 and 39.9°C (black broken lines). Fever in this study was recorded when rectal temperature was above 40°C. In experimentally infected animals, fever appeared between day 5 and 6 postinfection. (C) BTV RNA in blood samples of experimentally infected sheep and goats. Viral RNA was detected by qRT-PCR, and values are expressed as log_10_ copy number per μg of total RNA. Note that goats reached the highest level of BTV RNA in the blood. (D) Neutralizing antibodies toward BTV in experimentally infected animals. Sera were collected at the times indicated following experimental infection (time zero) and subjected to neutralization assays as indicated in Materials and Methods. Values shown are averages ± standard deviations and represent the log_10_ of the 50% endpoint (proportionate distance [PD]). Mock-infected goats and sheep (data shown in Fig. S2 in the supplemental material) did not show any clinical sign of bluetongue, maintained a physiological temperature throughout the experiment, and did not have any detectable BTV RNA or neutralizing antibodies.

Sheep infected with BTV-8_NET2006_ developed classic clinical signs of bluetongue, including fever (defined here as body temperature of >40°C), which started 4 to 5 days p.i., depression, anorexia, respiratory distress, increase in salivation, facial edema, and hyperemia of nasal and buccal mucosa (Fig. 2; see also Fig. S1 in the supplemental material, showing data for each individual animal). Overall, no major differences in clinical signs were observed between the three sheep breeds used in this study or between 8-month-old and 2-year-old Dorset poll sheep. In addition, no significant differences (*P* > 0.05) were observed in the levels of fever or the cumulative number of days with fever between all the sheep groups. However, one sheep in the mixed-breed infected group had to be euthanized because of onset of severe clinical signs. Consequently, the general and total clinical score of the infected mixed-breed group was higher than that of the other groups (Fig. 2A). In all the infected groups, BTV RNA in the blood peaked at about 5 days p.i. and then slowly decreased, although it remained detectable up to 4 weeks p.i., at which point the experiment was concluded (Fig. 2C; see also Fig. S1 in the supplemental material). Neutralizing antibodies were detected at day 7 p.i., peaked by day 14 p.i., and then remained essentially constant for the duration of the experiment (Fig. 2D).

On the other hand, goats after BTV-8_NET2006_ infection showed no clinical signs or fever throughout the duration of the experiment (28 days) (Fig. 2A and B). Differences in the body temperature between day 3 and 10 postinfection were statistically significant between goats and each of the groups of sheep described above (*P* < 0.0001). The onset of viremia in goats was delayed compared to that in infected sheep, peaking at 10 days postinfection. Average levels of BTV RNA in the blood were at least 10-fold higher in goats than in infected sheep between day 9 and 16 p.i., but overall the differences observed were not statistically significant due to individual variations (ANOVA *P* = 0.45) (Fig. 2C; see also Fig. S1 in the supplemental material). All mock-infected sheep and goat controls used in this study showed no clinical signs and remained negative for the presence of both viral RNA in the blood and neutralizing antibodies toward BTV (see Fig. S2 in the supplemental material).

### Influence of BTV strain and serotype on the clinical outcome of BTV infection.

**FIG 3:**
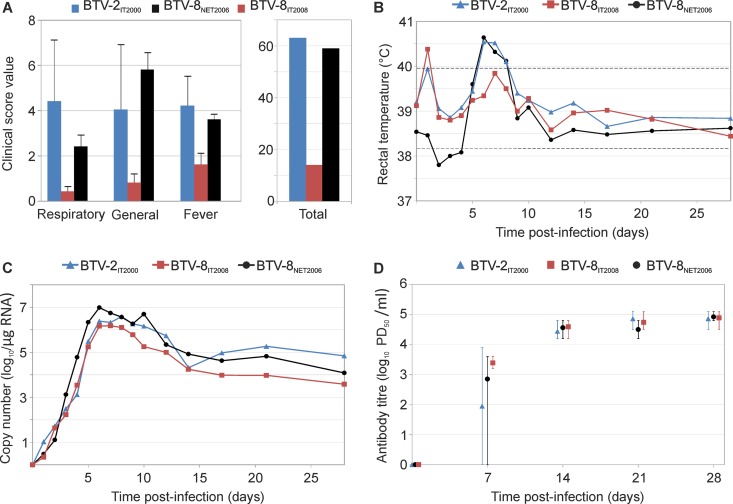
Virulence of BTV-2_IT2000_, BTV-8_NET2006_, and BTV-8_IT2008_. Clinical scores (A), rectal temperature (B), viremia (C), and neutralizing antibodies (D) of Sardinian sheep (*n* = 5 per group) infected with either BTV-2_IT2000_, BTV-8_NET2006_, or BTV-8_IT2008_. Descriptions of graphs in each panel are in the legend of Fig. 2. Note that experimental infections of sheep (Dorset poll, Dorset poll young, Sardinian, or mixed breed) and goats with BTV-8_NET2006_ and Sardinian sheep with BTV-2_IT2000_ or BTV-8_IT2008_ were carried out at the same time but are shown separately in Fig. 2 and 3 to facilitate the narrative. Consequently, the same sets of data for the Sardinian sheep infected with BTV-8_NET2006_ are shown both in Fig. 2 and 3. Fever and viremia data for each individual animal are shown in Fig. S3 in the supplemental material. Note that sheep infected with BTV-8_IT2008_ display very mild clinical signs, only a transitory fever, and lower levels of viremia than sheep infected with BTV-2_IT2000_ and BTV-8_NET2006_.

We also assessed the pathogenicity of different BTV serotypes, as well as different virus strains within a single serotype. The severity of disease observed in sheep inoculated with either BTV-2_IT2000_ or BTV-8_NET2006_ was largely equivalent, with both viruses inducing typical clinical signs observed in bluetongue (Fig. 3A). In contrast, animals infected with BTV-8_IT2008_ showed only a mild transitory fever but no other clinical signs (Fig. 3B; see also Fig. S3 in the supplemental material showing data for each individual animal). Excluding the temporary pyrexia displayed by some animals at day 1 p.i., BTV-8_NET2006_ and BTV-2_IT2000_ induced cumulatively 17 and 18 days of fever in their respective groups of infected sheep. In contrast, BTV-8_IT2008_ induced only 8 cumulative days of fever. Overall, we also observed that on average sheep infected with BTV-8_NET2006_ or BTV-2_IT2000_ displayed higher levels of fever than sheep infected with BTV-8_IT2008_, although differences were not statistically significant (ANOVA *P* = 0.17). BTV-8_IT2008_, BTV-8_NET2006_, and BTV-2_IT2000_ all induced similar levels of viremia (ANOVA *P* = 0.54) and neutralizing antibodies in infected sheep (Fig. 3C and D).

**FIG 4:**
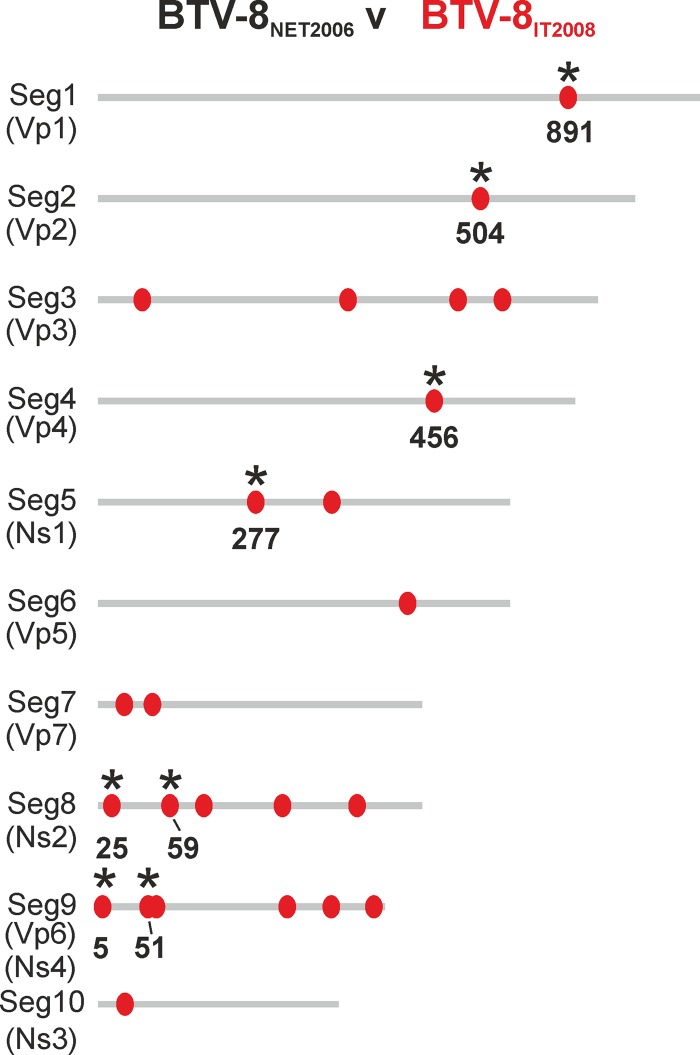
Genetic differences between BTV-8_NET2006_ and BTV-8_IT2008_. Schematic representation of the 10 genomic segments of BTV-8_NET2006_ and BTV-8_IT2008_. Mutations in BTV-8_IT2008_ compared to BTV-8_NET2006_ are indicated with red dots. Nonsynonymous mutations are highlighted with black asterisks, and the position of the mutated amino acid residue is shown. Note that the length of the schematic genome segments and the relative position of synonymous and nonsynonymous mutations in the cartoon are indicative only.

We next sequenced the complete genomes of BTV-8_NET2006_ and BTV-8_IT2008_ in order to determine the genetic basis for the different phenotypes of these two viruses. We detected a total of 24 nucleotide mutations between BTV-8_NET2006_ and BTV-8_IT2008_, including 16 silent mutations and 8 nonsynonymous mutations, leading to differences in the viral VP1, VP2, VP4, NS1, NS2, and VP6 proteins (Fig. 4).

### Effect of cell culture adaptation on BTV virulence.

**FIG 5:**
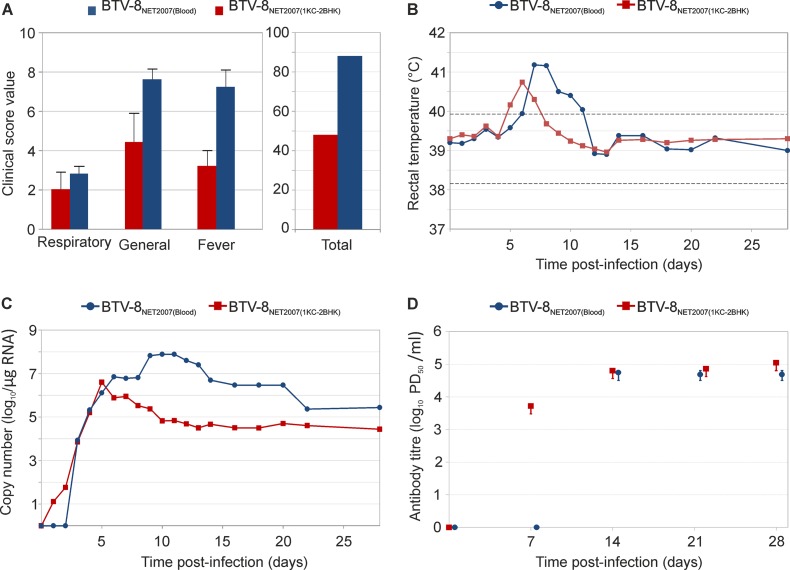
Experimental infection of Sardinian sheep with BTV-8_NET2007(blood)_ and BTV-8_NET2007(1KC-2BHK)_. Clinical scores (A), rectal temperature (B), viremia (C), and neutralizing antibodies (D) of Sardinian sheep (*n* = 5 per group) infected with either BTV-8_NET2007(blood)_ or BTV-8_NET2007(1KC-2BHK)_. Graphs in each panel have already been described in the legends of Fig. 2. Fever and viremia data for each individual sheep are shown in Fig. S4 in the supplemental material. Note that sheep infected with BTV-8_NET2007(blood)_ displayed more severe clinical signs and higher levels of fever and viremia than sheep infected with BTV-8_NET2007(1KC-2BHK)_.

Published reports suggest that, in some cases, infection of target species using blood directly from a naturally BTV-infected animal induces more severe clinical signs than tissue culture-adapted virus (20, 61). In the context of the experimental framework used in this study, we inoculated two groups of Sardinian sheep with either blood from a BTV-infected animal [BTV-8_NET2007(blood)_] or the same virus isolated in cell culture after a single passage in KC cells and two passages in BHK_21_ [BTV-8_NET2007(1KC-2BHK)_]. As assessed by qRT-PCR, the infected blood contained approximately 100-fold less viral RNA than the inoculum of BTV-8_NET2007(1KC-2BHK)_ (data not shown). Sheep infected with BTV-8_NET2007(blood)_ displayed a higher clinical score and reached statistically significant higher levels of fever (*P* = 0.01) than sheep inoculated with BTV-8_NET2007(1KC-2BHK)_ (Fig. 5A and B; see also Fig. S4 in the supplemental material). Sheep infected with BTV-8_NET2007(blood)_ displayed 27 cumulative days of fever as opposed to 16 shown by sheep infected with BTV-8_NET2007(1KC-2BHK)_. In addition, the levels of viral RNA in the blood were also consistently and considerably higher (10- to 1,000-fold; *P* = 0.018) in sheep infected with BTV-8_NET2007(blood)_ than those found in BTV-8_NET2007(1KC-2BHK)_-infected sheep (Fig. 5C; see also Fig. S4 in the supplemental material). Interestingly, viremia was delayed by 2 days in BTV-8_NET2007(blood)_-infected animals. In addition, we did not find neutralizing antibodies at 7 days postinfection in any of the sheep infected with BTV-8_NET2007(blood)_ (Fig. 5D). In contrast, all sheep infected with BTV-8_NET2007(1KC-2BHK)_ had BTV-neutralizing antibodies by day 7 p.i. No differences in the levels of neutralizing antibodies were found at later time points between sheep infected with BTV-8_NET2007(blood)_ and BTV-8_NET2007(1KC-2BHK)_. Thus, as proposed in other studies (20, 61), infection of sheep with BTV collected directly from infected animals and never passaged in tissue culture induced more severe clinical signs than the homologous virus passaged even minimally in tissue culture.

### BTV population diversity influences virulence.

Next, we aimed to link the phenotypic differences described above between sheep inoculated with BTV-8_NET2007(blood)_ and BTV-8_NET2007(1KC-2BHK)_ to genetic changes that might occur in the virus following cell culture adaptation. We analyzed the genomes of BTV-8_NET2007(blood)_ and BTV-8_NET2007(1KC-2BHK)_ by deep sequencing, using the same stocks utilized in the experimental infections described above. We also analyzed the intermediate viruses BTV-8_NET2007(1KC)_ and BTV-8_NET2007(1KC-1BHK)_. Furthermore, in order to test the reproducibility of the results obtained, we repeated in parallel the adaptation in KC and BHK_21_ cells of BTV-8_NET2007(blood)_ in an independent set of experiments. All together, we analyzed the full genome of 7 viral samples: BTV-8_NET2007(blood)_ and two independent isolates of BTV-8_NET2007(1KC)_, BTV-8_NET2007(1KC-1BHK)_, and BTV-8_NET2007(1KC-2BHK)_.

**FIG 6:**
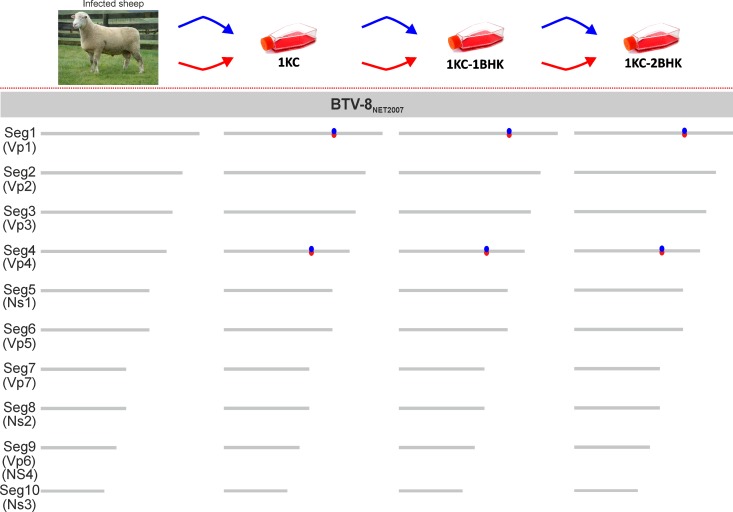
*In vitro* adaptation of BTV-8_NET2007(blood)_. The effects of adaptation *in vitro* of BTV-8_NET2007(blood)_ were assessed by comparing the genomic sequences of BTV-8_NET2007(blood)_ with the sequences of viruses isolated *in vitro* after passaging in Culicoides KC cells (1 passage) and two further passages in BHK_21_ cells. Schematic representation of the experiment is shown at the top of the figure. Two independent experiments (represented with blue or red arrows) were carried out, and sequences of the viral genome were obtained after each passage *in vitro*. The cartoon shows the schematic representation of individual genomic segments of BTV. Mutations found in the consensus sequences of the cell culture-passaged viruses are shown as red or blue dots, indicating the two independent experiments. Only two synonymous mutations were selected in Seg1 and Seg4 immediately after passage in KC cells in both independent experiments and were conserved after further passaging in BHK_21_ cells.

We found that the consensus sequences of BTV-8_NET2007(blood)_ and BTV-8_NET2007(1KC-2BHK)_ were identical, with the exception of two silent mutations in segments 1 (nucleotide 2756) and segment 4 (nucleotide 1431) (Fig. 6). Both point mutations were selected after the initial passage in KC cells and in both independent experiments.

RNA viruses, due to their high mutation rates, do not exist as a single genotype but as a complex of variants (also referred to as quasispecies), each possessing unique random mutations (74, 75). Consequently, we analyzed BTV-8_NET2007(blood)_ and the effect on its population diversity after passaging *in vitro* in KC and BHK_21_ cells.

**FIG 7:**
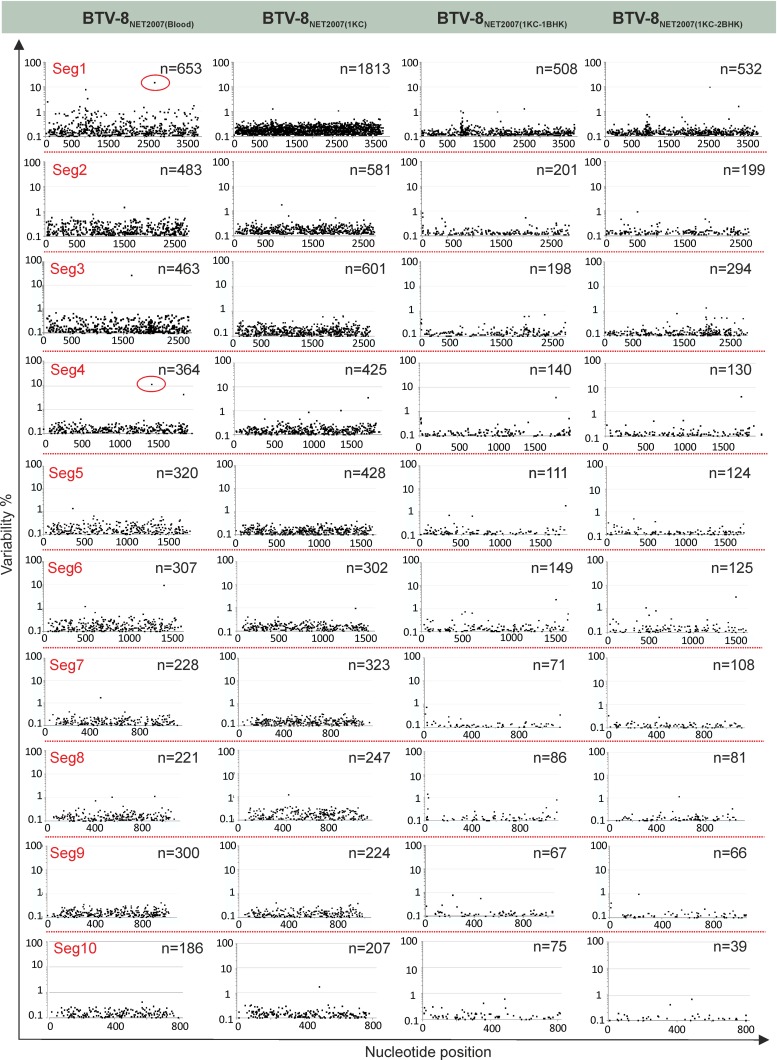
Viral population diversity of BTV-8_NET2007(blood)_ before and after isolation in cell culture. Changes in nucleotide diversity of BTV-8_NET2007(blood)_ amplified directly from the spleen of an infected sheep were compared with sequences of the same virus after isolation in KC and BHK_21_ cells. Differences were assessed by deep sequencing as described in Materials and Methods. Total reads of individual genome segments were mapped to consensus sequences, and single nucleotide polymorphisms (SNPs) were assigned above the arbitrary 0.1% frequency threshold. On the graph, each dot represents the percentage of nucleotide difference (*y* axis) from the consensus sequence of each nucleotide composing the individual genomic segments of the virus (*x* axis). The total number of variable nucleotides (>0.1%) for each sample is shown in the right corner of each plot. Dots circled in red in Seg1 and Seg4 of BTV-8_NET2007(blood)_ are those nucleotides that have been selected in the majority of the viral populations after passage *in vitro*.

**FIG 8:**
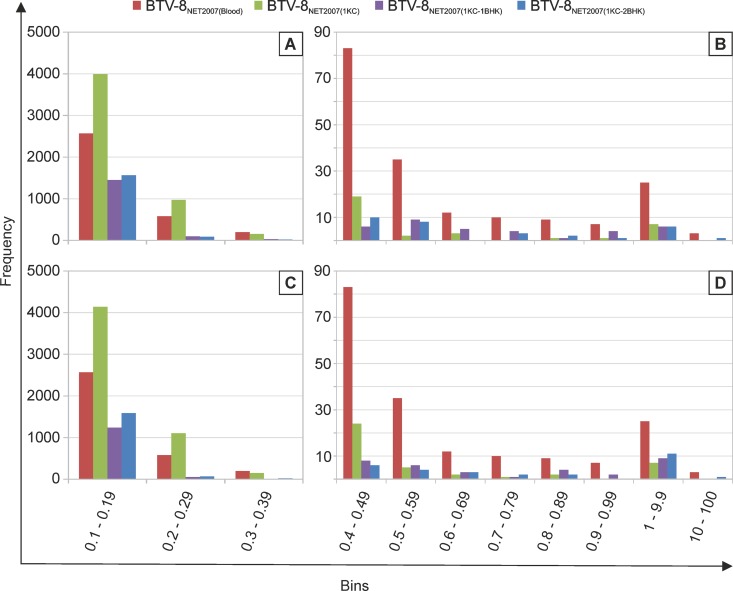
Frequency distribution of variable nucleotide in BTV-8_NET2007(blood)_, BTV-8_NET2007(1KC)_, BTV-8_NET2007(1KC-1BHK)_, and BTV-8_NET2007(1KC-2BHK)_. Histograms showing for each virus the number of nucleotides with percent variation falling within defined borders (“bins”). Panels A/B and C/D represent data from two independent experiments. Note that panels B and D have a different scale in the *y* axis from panels A and C, as the frequency of variants present in more than 0.4% of the total population was significantly lower than variants presented in panels A and C.

In Fig. 7, we have plotted the degree of variability at each nucleotide position of each genomic segment before and after passaging in cell culture. A nucleotide is plotted and is referred to as a “variant” if it represents at least 0.1% of the viral population. In general, the number of variants was higher in the virus before cell passaging, or after one passage in KC cells, than what observed even after a single passage in BHK_21_ cells. Interestingly, for 9 of the 10 segments in the first set of experiments, and for 8 of the 10 segments in the second set of experiments, the number of variable nucleotides was higher in the virus passaged once in KC cells than in the virus from blood before passage in cell culture. There was a larger number of variants with a frequency between 0.1 and 0.29% in BTV-8_NET2007(1KC)_, while the number of variants with a frequency of >0.4% was severalfold higher in BTV-8_NET2007(blood)_ (Fig. 8). The two silent mutations selected in the consensus sequence of BTV-8_NET2007(1KC-2BHK)_ were already present as high-prevalence variants in BTV-8_NET2007(blood)_ (14.9% for nucleotide 2756 of segment 1 and 10.4% for nucleotide 1431 of segment 4) (dots circled in red in Fig. 7). On the other hand, other variants present with a frequency of about 10% in segment 3 and segment 6 were not selected after passage *in vitro*. Essentially, the same results were obtained in the two independent sets of experiments.

## DISCUSSION

Most infections of susceptible hosts by pathogenic viruses result in clinical manifestations that can vary greatly in their severity. For some viruses, such as avian influenza virus, for example, low and highly virulent strains are distinguishable by clear genotypic differences (76). Nevertheless, in some circumstances, even infection of susceptible hosts with highly pathogenic viruses can result in mild or unapparent clinical symptoms.

Bluetongue is a disease characterized by a highly variable clinical spectrum (21–24). Understanding the basis for this variability is complicated by the fact that BTV exists in nature as many diverse strains representing different serotypes, topotypes, and reassortant viruses often cocirculating in the same geographical area. In addition, BTV can infect a variety of ruminant species, each with different genetic and immunological backgrounds. Furthermore, BTV is transmitted by different species of Culicoides in diverse ecological contexts. There have been several studies concerning naturally occurring bluetongue or experimentally induced disease, clearly indicating that factors related to both the mammalian host and the virus can influence the outcome of BTV infection (55). However, it is not always straightforward to compare data from different studies. Thus, the weight given to different host or virus factors in determining the clinical outcome to BTV infection can differ in heterogeneous ecological or experimental settings.

In this study, we dissected both host and virus factors that can affect the clinical outcome of BTV infection. The use of a uniform experimental framework has allowed us to rigorously interrogate both experimental questions addressed in past studies (55), as well as to explore hitherto unanswered questions. First of all, as suggested previously (24, 32–36), we confirmed that while both sheep and goats are fully susceptible to BTV (in this case BTV-8) infection, the former are more susceptible than goats and more likely to develop clinical disease. The levels of viremia in BTV-infected goats were not different from (if anything, higher than) those observed in infected sheep. These data confirm that BTV is able to replicate to high levels in goat tissues but cellular damage, either induced by the virus or the host immune responses, does not likely occur. We do not know if goats would be more susceptible to disease if we had used higher infectious doses. We have used 2 × 10^6^ PFU of BTV in our experimental infections, and this is likely far more infectious virus than is transmitted in nature by infected midges. In addition, studies in sheep using as little as 10^1.4^ TCID_50_ were able to induce infection in this animal species (66). In two previous studies, also using BTV-8 isolates from the Netherlands, some of the experimentally infected goats developed mild clinical signs, fever, and viremia (34, 36). However, in both studies, goats were infected intravenously (34, 36), and in one of them animals were infected at day 62 of gestation (36). Another study used BTV-4, which was isolated in embryonated chicken eggs and passaged seven times in BHK_21_. Only 1 of 11 goats (of two different breeds) infected with this virus showed transient pyrexia, but at the same time 10 of 12 inoculated sheep did not show fever or signs of disease either (32). Thus, this study confirmed that the mammalian host species is certainly one of the main factors that determine the clinical outcome of BTV infection.

We did not find major differences in the susceptibility of sheep breeds from the Mediterranean area (Sardinian and Italian mixed breed) and Northern European breeds (Dorset poll) to bluetongue, despite their distinct geographical, historical, and breeding backgrounds (47). Thus, variations in the susceptibility to bluetongue of different sheep breeds might not be as pronounced as originally thought. It is also important to stress that bluetongue itself was first discovered in European breeds imported into South Africa (77). Those breeds showed a higher susceptibility to bluetongue than local animals, although the influence of herd immunity on the latter could have also played a role. It is therefore difficult to weigh the influences of the host's genetic background, previous BTV exposure, or the insect vector on the susceptibility to the disease in that particular context.

We have also analyzed the influence of divergent viral serotypes, and closely related but distinct strains within the same serotype, on the clinical outcome of bluetongue. BTV-8_NET2006_ is considered to be a highly pathogenic virus (both in terms of morbidity and mortality) and the cause of one of the largest outbreaks of bluetongue in history (48–51). However, in our experimental setting, we did not find any difference in virulence between BTV-8_NET2006_ and another serotype, such as BTV-2_IT2000_, which was isolated in Italy in the year 2000 from a naturally occurring case of bluetongue in sheep. Another study, comparing the virulence of BTV-1 isolated from Algeria and a 2006 isolate of BTV-8 from Belgium, concluded that the former was more virulent than the latter (78). Although in that particular study the cell culture passage history was not described and viruses were inoculated subcutaneously, it appears that the overall data suggest that in itself BTV-8_NET2006_ is not necessarily more virulent than other BTV serotypes, such as BTV-2 or BTV-1, that have been circulating in Europe in the last decade. It is likely that other factors, such as the rapid spread of the infection to an extremely large number of fully susceptible and naive hosts (never previously exposed even to heterologous BTV serotypes), contributed to the number of severe cases of disease observed during the Northern European outbreak caused by this strain of BTV.

The BTV-8_NET2006_ strain was isolated from samples collected at the beginning of the European outbreak of this virus. Since the original cases identified in 2006 in central Europe, BTV-8 moved in subsequent years toward several surrounding geographical areas (including southward). Interestingly, in Northern Italy and in Sardinia, BTV-8 (termed in this study BTV-8_IT2008_) was detected only at the serological level in a few animals, but it was not associated with clinical disease (G. Savini, personal communication). We showed conclusively in our study that BTV-8_IT2008_ was less virulent than BTV-8_NET2006_. BTV-8_IT2008_ accumulated several nonsynonymous mutations in structural and nonstructural proteins (including VP1, VP2, NS1, and NS2) already implicated in attenuation of tissue culture-adapted BTV-2, BTV-4, and BTV-9 (57). Thus, this study formally proves the appearance of less virulent strains during a BTV outbreak. The comparative smaller number of severe cases of bluetongue in areas where it is endemic might depend upon several factors, including the levels of herd immunity, the decrease in virulence of circulating BTV strains, and, possibly, the long-term selection of genetically resistant individual animals.

Finally, we further investigated the observation that experimental infection of sheep with blood collected from naturally occurring cases of bluetongue appears to induce, in general, more severe clinical cases compared to the disease induced in sheep infected with viruses isolated in tissue culture or embryonated eggs (20, 61). Indeed, we have confirmed in our experimental framework that sheep inoculated with BTV-8_NET2007(blood)_ displayed a more severe disease and higher levels of viremia than those infected with the virus isolated in cell culture [BTV-8_NET2007(1KC-2BHK)_]. It is unlikely that factors present in the infected blood could be the cause of more severe clinical signs in sheep. Importantly, the highest levels of fever and the most severe clinical signs in sheep infected with BTV-8_NET2007(blood)_ were observed between days 6 and 11 p.i., when the levels of BTV in the blood were at their highest.

Virus passaging in tissue culture can lead to adaptive changes in the viral genotype that could in turn affect viral virulence. However, we found only 2 synonymous mutations between the consensus sequence of BTV-8_NET2007(blood)_ and the cell culture-isolated virus BTV-8_NET2007(1KC-2BHK)_. Both mutations were present in approximately 10% of the variants of BTV-8_NET2007(blood)_, and interestingly they were both selected in two independent experiments. It is possible that these silent mutations in some way affect viral virulence. In addition, the sequencing methods used did not cover the noncoding regions of each segment, and therefore we may have also missed other important mutations. However, overall there appears to be very little (or no variation at all) at the consensus sequence level (at least for BTV-8) of viruses isolated from blood or minimally passaged in cell culture. RNA viruses have the highest error rates (10^−4^ to 10^−6^ per nucleotide site per genome replication) of any microorganism due to their RNA-dependent RNA polymerase lacking proofreading activity during RNA synthesis (79, 80). As such, RNA viruses exist as a population of variants, genetically closely related but distinct from their consensus sequence. It is rational to argue that the opportunity to quickly adapt and generate diverse viral populations is critical for the survival of RNA viruses (74) in the face of selective pressures, including the innate and adaptive antiviral responses of the host. For example, poliovirus mutants with a high-fidelity polymerase (and thus low population diversity) display an attenuated phenotype in mice, despite possessing identical consensus sequences to the virulent wild-type viruses (81–83).

We found that BTV-8_NET2007(blood)_ contained the largest number of high-frequency variants. However, when BTV-8_NET2007(blood)_ was passaged in insect KC cells, the resulting viral population [BTV-8_NET2007(1KC)_] showed the overall highest number of variants, even higher (∼ 60%) than those in the blood before tissue culture isolation. A severe genetic bottleneck was observed after viral passaging in mammalian BHK_21_ cells, with the resulting viruses [BTV-8_NET2007(1KC-1BHK)_ and BTV-8_NET2007(1KC-2BHK)_] showing the smallest degree of variability.

These data suggest that BTV virulence is affected not only by changes in the viral proteins selected at the consensus level but also by the genetic variability of the population as a whole. This hypothesis is also supported by previous observations made in a limited number of genes before the advent of deep sequencing (84, 85). In a study that analyzed segment 2 of a virulent strain of BTV-1, Gould and Eaton (84) showed that the consensus sequence did not change after a single passage in tissue culture that resulted in viral attenuation. In addition, Bonneau and colleagues (85) showed that the number of variants observed in segment 2 and 10 of plaque-purified BTV-10 increased during transmission of the virus between ruminants and insect vectors but without changes to the consensus sequence.

Thus, “flat” populations containing a relatively small number of variants appear to be less virulent than more variable populations.

In addition, our data also suggest that Culicoides cells might function as a natural source of new BTV variants. BTV is an arbovirus and as such must adapt rapidly to replicate in hosts as different as a warm-blooded mammal and insects. An increased variability of replication in Culicoides cells might allow BTV to adapt faster to different selective pressures present in the invertebrate and vertebrate hosts. These data also reinforce the notion that it is critical to avoid the use of modified live vaccines that induce even transient viremia in vaccinated animals. The transmission of vaccine strains in the Culicoides population might then lead to the emergence of “new” strains with the potential to revert to their original phenotype.

Our study has not taken into consideration factors related to the invertebrate host (e.g., species and sites and number of “infectious” bites) that could affect BTV pathogenesis. The insect host certainly plays a role in modulating the interaction between virus and the mammalian host, as some studies are beginning to suggest (86). It is possible that transmission of BTV by different species of Culicoides, in different geographical areas, could influence the pathogenesis of bluetongue in different ways. This is an exceedingly important area of research that will need to be addressed in the coming years.

## Supplementary Material

Supplemental material
