# Supplementary material for: Virus and Host Factors Affecting the Clinical Outcome of Bluetongue Virus Infection
Source: J Virol. 2014 Sep;88(18):10399–411. doi: 10.1128/JVI.01641-14 (PMC4178883; doi:10.1128/JVI.01641-14)
Supplement: Supplemental material [file supp_88_18_10399__index.html]

Virus and Host Factors Affecting the Clinical Outcome of Bluetongue Virus Infection — Supplemental material 

# Virus and Host Factors Affecting the Clinical Outcome of Bluetongue Virus Infection

## Supplemental material

**Files in this Data Supplement:**

- Supplemental file 1 -

  Table S1 (Clinical score index.)

  Fig. S1 (Experimental infection of goats and different sheep breeds with BTV-8NET2006.)

  Fig. S2 (No clinical signs, fever, viremia or neutralizing antibodies in mock-infected control goats and sheep.)

  Fig. S3 (Virulence of BTV-2IT2000 , BTV-8NET2006, and BTV-8IT2008)

  Fig. S4 (Experimental infection of sheep with BTV-8NET2007(blood) and BIV-8NET2007(1KC-2BHK).)

  PDF, 5.7M
