# Supplementary material for: Virus and Host Factors Affecting the Clinical Outcome of Bluetongue Virus Infection
Source: J Virol. 2014 Sep;88(18):10399–411. doi: 10.1128/JVI.01641-14 (PMC4178883; doi:10.1128/JVI.01641-14)
Supplement: Supplemental material [file JVI.01641-14_zjv999099491so1.pdf]

**Supplementary Table S1.** Clinical score index used in this study.

| | | Symptom (points) | Absent (0) | Mild (1) | Moderate (2) | Severe (3) |
|---|---|---|---|---|---|---|
| General | Sensory | **Depression** | Responsive (sheep is alert and responsive to environmental stimuli) | Sheep is listless | Apathy, sheep react only if stimulated, separating from the flock | Prostration, not responsive to stimuli |
| | | **Anorexia** | Normal feeding | Not responding when food is provide | Only minimal feeding during the day | No eating at all |
| | Facial Lesions | **Salivation** | No secretion | Lower jaw wet from saliva | Sheep is actively secreting | Hypersalivation |
| | | **Facial oedema** | Absence of oedema | Local oedema | Multiple oedemas localized in different regions | Marked facial oedema, “bottle jaw” (submandibular oedema) |
| | | **Muco-cutaneous and oral lesions** | No lesions | Hyperemia and inflammation labial mucosa/gums, blisters appear on oral mucosa | Petechial and ecchymotic hemorrhages in the oral mucosa/oral erosions | Confluent erosions oral mucosa/ulcers |
| | | **Ocular discharge** | Absence of ocular clinical signs | Lacrimation/ red mucous membranes | Discharge/conjunctivitis | Cataractal, white haze, conjunctivitis |
| | Feet lesions | **Dermal and hoof coronet lesions** | No lesions/normal derma | Reddening of skin above coronary band, sheep doesn’t show any difficulty of movements | Coronitis, warm lower limbs and hooves (body mass is moved to other legs) | Severe lameness, stiff gait, hunched appearance/sheep may kneel/lying down/crippled |
| Respiratory symptoms | | **Lower airway distress** | Normal respiratory rate* (≤ 40 breath/min) | Slightly increased respiratory rate* (41-60) and/or abnormal breathing sounds | Increased respiratory rate* (61-100) and/or clear abdominal breathing | Strongly increased respiratory rate* (>100) and/or rapid abdominal breathing and/or mouth breathing/abnormal breathing sound (froth) |
| | | **Nasal discharge** | Absence of ocular/nasal clinical signs | Intermittent serous (clear) to mucoid (gray and cloudy) discharge/lacrimation | Persistent mucoid (gray and cloudy) to purulent (thick and yellowish/green) discharge | Purulent (thick and yellowish/ green) to bloody discharge |
| | | **Upper airway distress** | Normal breathing | Rare coughing/sneezing (only during physical activity) | Frequent coughing/sneezing (also at rest) | Frequent coughing/sneezing at rest with prolonged episodes |
| Fever | | | Body temperature below 40°C | Body temperature equal or above 40°C | Body temperature equal or above 41°C | / |
| Veterinary intervention | | | | | | **8 Points** |
| Animal Death | | | | | | **8 Points** |

## Supplementary Figure S1

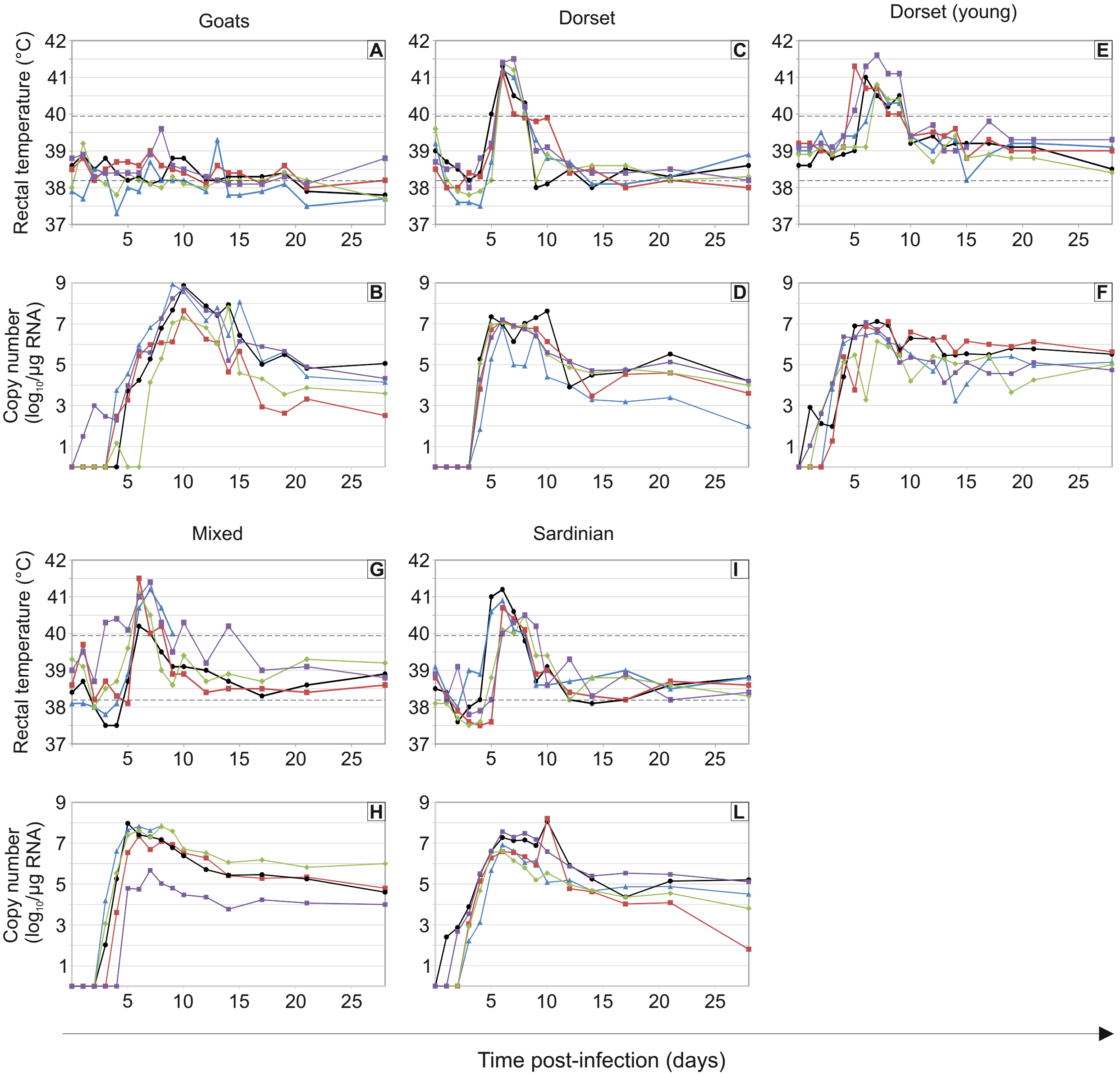


**Supplementary Figure S1. Experimental infection of goats and different sheep breeds with BTV-8$_{NET2006}$.** Body (rectal) temperature, and levels of BTV RNA in blood of experimentally infected sheep and goats. Panels show the data of each experimentally infected animal within the indicated groups. Physiological temperature in sheep ranges between 38.3 and 39.9°C and are shown as broken lines.

## Supplementary Figure S2

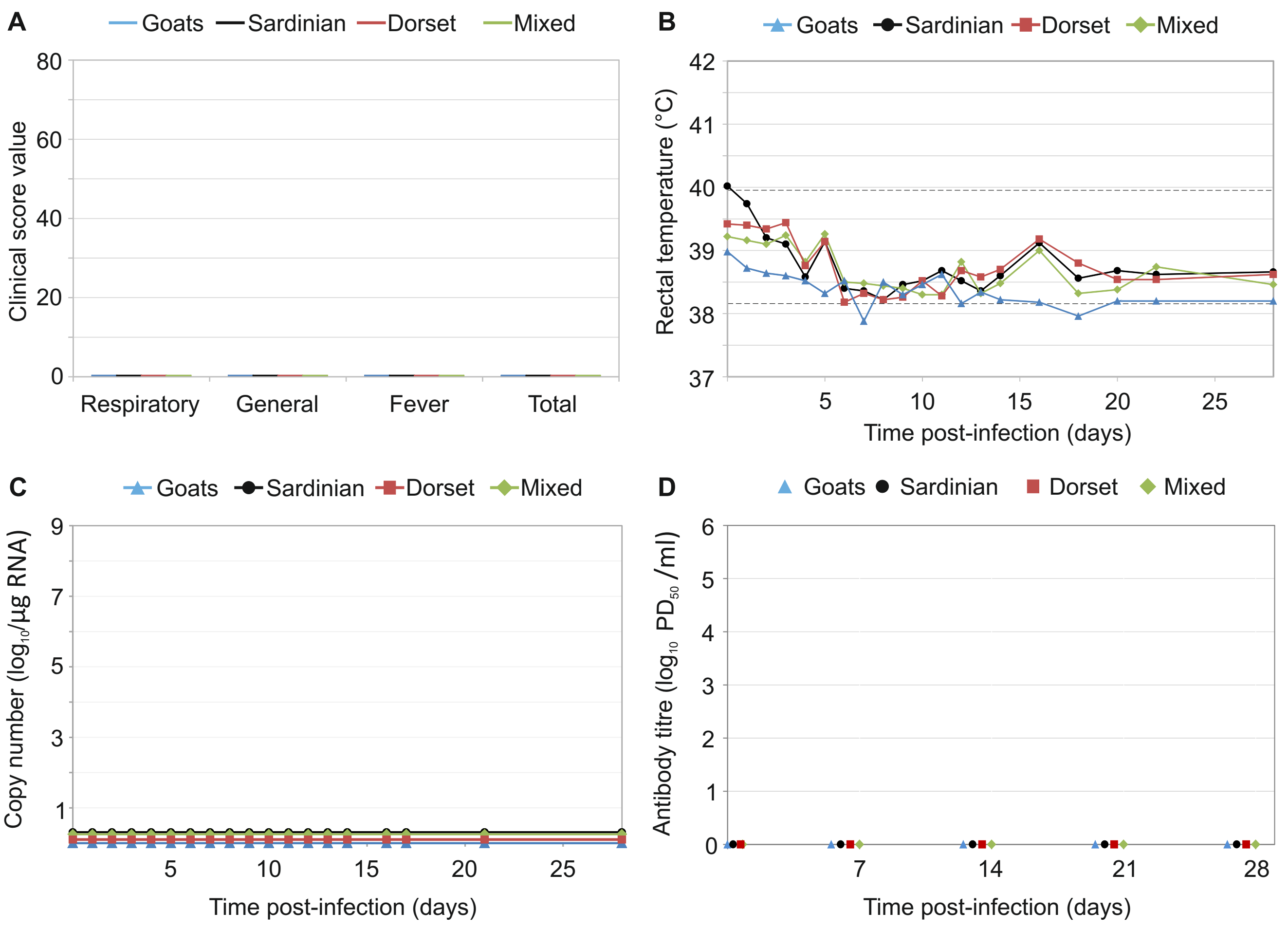


**Supplementary Figure S2. No clinical signs, fever, viremia or neutralizing antibodies in mock-infected control goats and sheep.** Data obtained in mock-infected goats and sheep used in this study. **A**. Clinical scores. **B.** Body (rectal) temperature (average per group). Physiological temperature in sheep ranges between 38.3 and 39.9°C and are shown as broken lines. **C.** BTV RNA in blood. **D**. Neutralizing antibodies towards BTV.

## Supplementary Figure S3

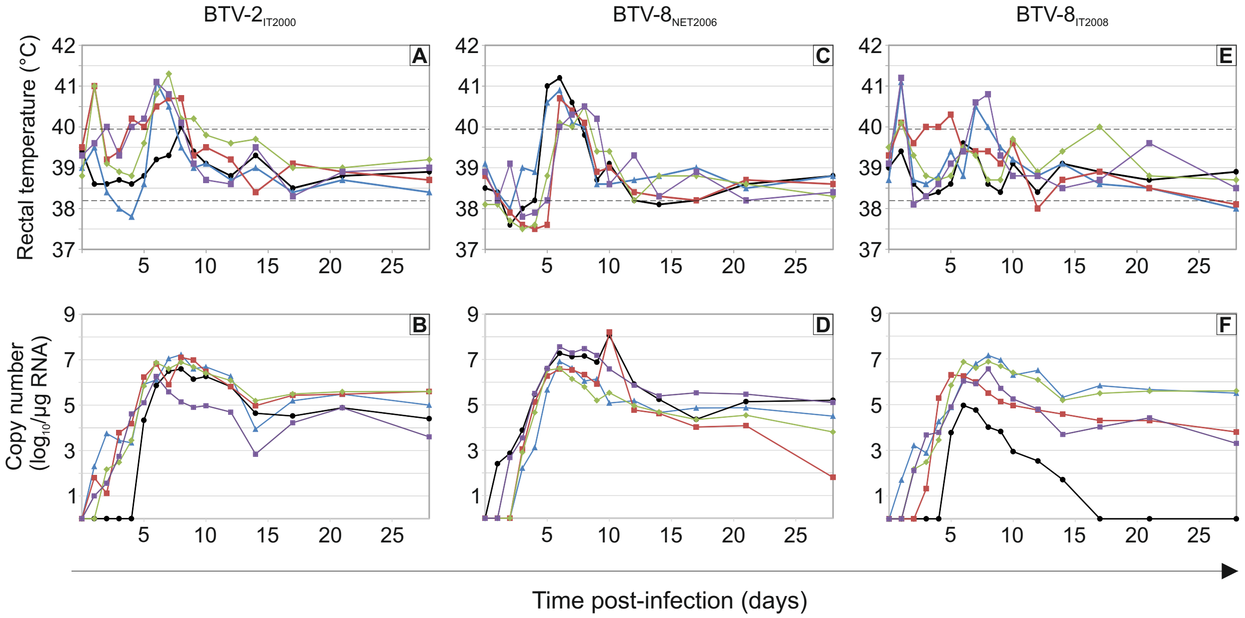


**Supplementary Figure S3. Virulence of BTV-2$_{IT2000}$, BTV-8$_{NET2006}$ and BTV-8$_{IT2008}$.** Body (rectal) temperature, and levels of BTV RNA in blood of experimentally infected sheep and goats. Panels show data obtained in each experimentally infected sheep within the indicated groups infected with either BTV-2$_{IT2000}$, BTV-8$_{NET2006}$ or BTV-8$_{IT2008}$. Physiological temperature in sheep ranges between 38.3 and 39.9$^{o}$C and are shown as broken lines.

## Supplementary Figure S4

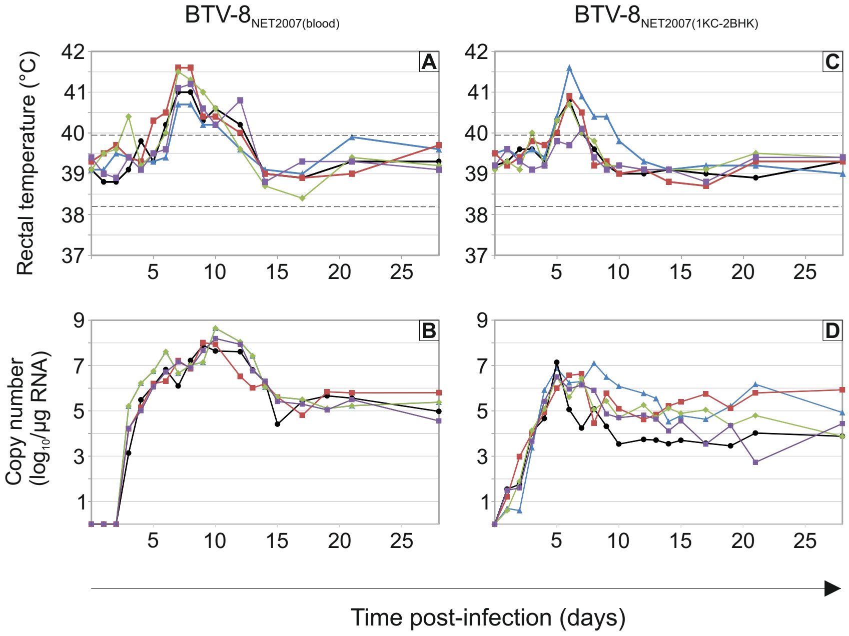


**Supplementary Figure S4. Experimental infection of sheep with BTV-8$_{NET2007(blood)}$ and BTV-8$_{NET2007(1KC-2BHK)}$.** Body (rectal) temperature, and levels of BTV RNA in blood of experimentally infected sheep and goats. Panels show data obtained in each experimentally infected sheep within the indicated groups infected with either BTV-8$_{NET2007(blood)}$ or BTV-8$_{NET2007(1KC-2BHK)}$. Physiological temperature in sheep ranges between 38.3 and 39.9$^{o}$C and are shown as broken lines.
